# Breastfeeding self-efficacy of women using second-line strategies for healthy term infants in the first week postpartum: an Australian observational study

**DOI:** 10.1186/1746-4358-8-18

**Published:** 2013-12-20

**Authors:** Frances Keemer

**Affiliations:** 1 Queensland University of Technology, Victoria Park Road, Kelvin Grove, Queensland 4059, Australia

**Keywords:** Breastfeeding self-efficacy, Breastfeeding challenge, Term infant, Cup feeding, Syringe feeding, Finger feeding, Bottle feeding, Nipple shield, Woman-centred care

## Abstract

**Background:**

Breastfeeding self-efficacy (BFSE) supports breastfeeding initiation and duration. Challenges to breastfeeding may undermine BFSE, but second-line strategies including nipple shields, syringe, cup, supply line and bottle feeding may support breastfeeding until challenges are resolved. The primary aim of this study was to examine BFSE in a sample of women using second-line strategies for feeding healthy term infants in the first week postpartum.

**Methods:**

A retrospective self-report study was conducted using the Breastfeeding Self-Efficacy Scale - Short Form (BSES-SF), demographic and infant feeding questionnaires. Breastfeeding women who gave birth to a singleton healthy term infant at one private metropolitan birthing facility in Australia from November 2008 to February 2009 returned anonymous questionnaires by mail.

**Results:**

A total of 128 (73 multiparous, 55 primiparous) women participated in the study. The mean BSES-SF score was 51.18 (Standard deviation, SD: 12.48). The median BSES-SF score was 53. Of women using a second-line strategy, 16 exceeded the median, and 42 were below. Analyses using Kruskal-Wallis tests confirmed this difference was statistically significant (H = 21.569, p = 0.001). The rate of second-line strategy use was 48%. The four most commonly used second-line strategies were: bottles with regular teats (77%); syringe feeding (44%); bottles with wide teats (34%); and nipple shields (27%). Seven key challenges were identified that contributed to the decision to use second-line strategies, including: nipple pain (40%); unsettled infant (40%); insufficient milk supply (37%); fatigue (37%); night nursery care (25%); infant weight loss > 10% (24%); and maternal birth associated pain (20%). Skin-to-skin contact at birth was commonly reported (93%). At seven days postpartum 124 women (97%) were continuing to breastfeed.

**Conclusions:**

The high rate of use of second-line strategies identified in this study and high rate of breastfeeding at day seven despite lower BFSE indicate that such practices should not be overlooked by health professionals. The design of this study does not enable determination of cause-effect relationships to identify factors which contribute to use of second-line strategies. Nevertheless, the significantly lower BSES-SF score of women using a second-line strategy highlights this group of women have particular needs that require attention.

## Background

During the first week postpartum many breastfeeding challenges can occur. Maternal challenges include nipple shape, pain, or damage, perceived insufficient milk supply and engorgement [1]. Further maternal challenges include birth related pain, type of pain relief used in labour, and fatigue [2,3]. Infant challenges can include sleepiness, poor or disorganized suck, demanding behaviour, dehydration evidenced by excessive birth weight loss, and hypoglycaemia [1,4-7]. First-line strategies include skin-to-skin contact at birth and unrestricted infant led breastfeeding that aim to prevent or reduce challenges [4,8-10]. Failure to facilitate early breastfeeding of healthy term infants can result in dehydration requiring admission to Special Care Nurseries for fluid and blood glucose management with invasive and painful procedures [6,7,11]. Therefore, second-line strategies are a vital resource to support breastfeeding women and infants during challenges, particularly in the first week postpartum.

Second-line strategies include a range of devices and techniques that compensate for challenges whilst women continue to pursue their breastfeeding goal [1], and provide a means to maintain infant nutrition/hydration temporarily. Cup feeding, syringe feeding, finger feeding, supply lines (supplemental nursing systems), bottles with teats, and nipple shields are examples of second-line strategies. With the exception of nipple shields, all second-line strategies can be used for either breast milk or breast milk substitutes.

Breastfeeding self efficacy (BFSE) is an important focus of interventions designed to support breastfeeding women, enable success of first and second-line strategies, and achieve successful breastfeeding outcomes. Numerous studies have identified that BFSE is an important factor in breastfeeding outcomes including duration [12-14]. One study has identified that feeding method as planned is a predictor of BFSE [12]. Other variables known to predict BFSE levels include breastfeeding intention, maternal education, satisfaction with labour pain relief, support from other women with children, perceptions of breastfeeding progress, and maternal anxiety [12,13]. While these relationships have been observed in a growing number of studies, the factors which contribute to BFSE are not clearly understood. For example, one study has reported that women with high or low confidence levels both experience breastfeeding challenges [15]. Moreover, although several studies present reasons for cessation of breastfeeding, including specific maternal and/or infant challenges, the relationship between these challenges and BFSE is unclear [3,16,17].

Little is known about the relationship between BFSE and use of second-line strategies during the first week postpartum. The primary purpose of this research was to measure BFSE retrospectively across the first seven days postpartum, and to examine the relationship between use of second-line strategies in the first week postpartum and BFSE.

## Methods

### Study design and setting

The study design was a quantitative, observational retrospective self-report survey. The research was conducted at one Australian metropolitan UnitingCare Health private birthing facility, located in Brisbane.

### Participants

**Figure 1:**
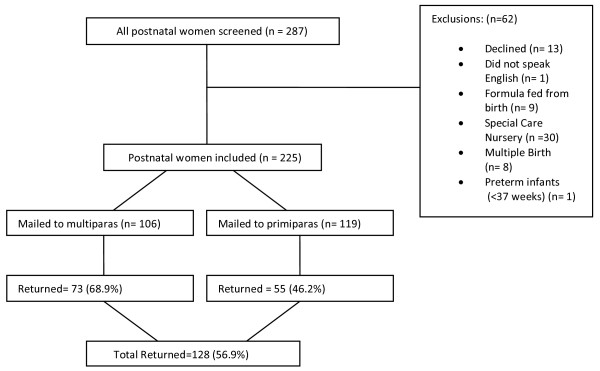
Response rate, parity and reasons for exclusion.

Participants were identified through systematic chart review of all current inpatients, from 1 November 2008 to 27 February 2009. Following the birth of their healthy term singleton infant, and initiating any breastfeeding, participants were invited to complete a questionnaire mailed to their home by day seven. Length of postnatal inpatient stay at this facility was four/five days. Figure 1 shows women screened for inclusion, reasons for exclusion, and response rate of both primiparous and multiparous women.

### Ethics

Ethical approval was granted from UnitingCare Health Human Research Ethics Committee (200837), and Queensland University of Technology Human Research Ethics Committee (0800000784). No identifying information was contained on the questionnaires and consent was implied by return of the questionnaire [18].

### Measures

Women were asked to respond to a range of questions regarding demographic, birthing, feeding variables, and the 14 item Breastfeeding Self Efficacy Scale - Short Form (BSES-SF), yielding nominal and interval data. Permission to use the scale was granted by the owner. International studies have measured BFSE, with repeated reliability and validity, including one Australian study [19-26]. The timing of BFSE measurement varies in the literature and includes antenatal, in-hospital, and up to six months postpartum measurement. The BSES-SF tool is designed for women to reflect on the previous 24 hours when choosing their responses. Whilst providing a precise snapshot of BFSE, it may not reflect day-to-day lactogenesis II changes, particularly during the first week postpartum. Therefore, data was collected at day seven, and all responses were the participant’s retrospective view of the first seven postnatal days. In this study, internal consistency of the BSES-SF with minor retrospective changes was confirmed with Cronbach’s alpha 0.93. The BSES-SF provided interval data with scores ranging from 14 (lowest) to 70 (highest). The eight second-line strategies were grouped together to form a single variable representing the number of women who used second-line strategies for the purpose of analyses. The group of second-line strategies was developed from the literature. A panel of 2 midwives and 2 mothers was requested to review the whole questionnaire to comment on its face validity (these mothers did not participate in the survey). Suggestions received from this panel were incorporated into the final questionnaire.

### Data management and analysis

All questionnaires were checked for missing data. One respondent omitted a page, and two others had other missing data but this was minimal so their available data were included in the analysis. Seven respondents omitted an item in the BSES-SF, and these were not included in analysis. Data were analysed using SPSS version 16.0. Descriptive statistics were used to describe the demographic profile of the respondents, present the rates of use of second-line strategies and the reasons for their use. Due to large variances, the non-parametric Kruskal-Wallis Analysis of Variance [27,28] was used to test median BSES-SF score for the two groups. The probability level chosen for significance testing was p-value < 0.05.

## Results

### Demographic profile

Of 225 women identified for inclusion (Figure 1), 128 returned questionnaires (73 multiparous and 55 primiparous), providing a 57% response rate. The majority of women were aged between 30 and 39, were married, had combined annual incomes over AU$100 000, and university or post-graduate level education (Table 1).

**Table 1:** **Sociodemographic and biomedical characteristics of sample (n** = **128)**

**Sociodemographic**	**n (%)**	
Age		
20–24	2 (2)	
25–29	22 (17)	
30–34	53 (41)	
35–39	42 (33)	
40–44	8 (6)	
Missing	1 (1)	
Marital status		
Living with partner	14 (11)	
Married	114 (89)	
Combined annual income AU$		
Below 50 000	2 (2)	
50 000 – 75 000	17 (13)	
75 000 – 100 000	27 (21)	
Over 100 000	77 (60)	
Missing	4 (4)	
Education		
Year 10	4 (3)	
Year 12	14 (11)	
Technical and further education (TAFE)	16 (13)	
University	58 (45)	
Postgraduate or higher	36 (28)	
**Biomedical**		
Birth mode		
Normal vaginal	53 (41)	
Vacuum extraction or forceps	15 (12)	
Caesarean	60 (47)	
Type of pain relief in labour		
None	9 (7)	
Nitrous oxide	34 (27)	
Pethidine	12 (9)	
Epidural/spinal	100 (79)	
General anaesthetic	3 (2)	
Don’t know	1 (1)	
Missing data	1 (1)	
Parity		*Previously breastfed ≥ 6mths (% multiparas)*
Primipara – 1	55 (43)	-
Multipara – 2	48 (38)	45 (94)
Multipara – 3	21 (16)	19 (90)
Multipara – 4	4 (3)	1 (25)
Infant birth weight (n = 125)	*Male Female*	
2500–2999 g	4	3
3000–3499	18	23
3500–3999	36	24
4000–4499	10	5
4500–4999	0	2

The majority of infants weighed between 3000 g and 4000 g, and just over half the women were multiparous, with a history of breastfeeding a previous infant for more than six months (Table 1). Almost all (93%) women reported skin-to-skin contact with their infant at birth. Of 128 women who began breastfeeding, four ceased breastfeeding by seven days. More than three quarters of the women reported using spinal or epidural pain relief during the delivery. Forty-seven percent gave birth by Caesarean section (60/128), 97% of these were planned (58/60).

### Use of second-line strategies

The frequency of second-line strategy use and parity is shown at Table 2. Sixty-two women reported using one or more second-line strategies. ‘Bottle with regular teat’ was the single most commonly used second-line strategy. However when combined, syringe feeding, finger feeding with a syringe, and finger/tube and syringe feeding (n = 44) were most frequently used. The ‘cup’ strategy was more frequently used by primiparous women, but there were only 12 instances of use in total. The nipple shield strategy was utilized by similar proportions of primiparous and multiparous women, with 17 instances of use.

**Table 2:** Second-line strategies and parity

**Frequency of use – second-line strategies**			**Primiparous**	**Multiparous**	**Total**
			**n** = **36**	**n** = **26**	**n** = **62**
	Bottle	Once only	5	4	9
	Regular	Up to 5 times	8	7	15
	Teat n = 38	More than 5 times	10	4	14
		*Knowledge*	*44*	*69*	113*(91.9)
		*Preference*	*0*	*3*	*3(2.4)*
	Bottle	Once only	1	4	5
	Wide teat	Up to 5 times	4	2	6
	n = 22	More than 5 times	8	3	11
		*Knowledge*	*39*	*58*	97*(78.9)
		*Preference*	*1*	*0*	*1(0.8)*
	Syringe only	Once only	6	5	11
	n = 27	Up to 5 times	5	6	11
		More than 5 times	4	1	5
		*Knowledge*	*24*	*48*	72*(58.5)
		*Preference*	*0*	*1*	*1(0.8)*
	Syringe+	Once only	2	0	2
	Finger feeding	Up to 5 times	3	3	6
	n = 13	More than 5 times	5	0	5
		*Knowledge*	*11*	*15*	26*(21.1)
		*Preference*	*2*	*2*	*4(3.3)*
	Syringe + tube+	Once only	0	0	0
	Finger feeding	Up to 5 times	2	0	2
	n = 4	More than 5 times	2	0	2
		*Knowledge*	*8*	*14*	22*(17.9)
		*Preference*	*1*	*0*	*1(0.8)*
	Cup	Once only	4	2	6
	n = 12	Up to 5 times	5	1	6
		More than 5 times	0	0	0
		*Knowledge*	*14*	*30*	44*(35.8)
		*Preference*	*1*	*0*	*1(0.8)*
	Supply line	Once only	0	0	0
	n = 1	Up to 5 times	0	0	0
		More than 5 times	1	0	1
		*Knowledge*	*6*	*13*	19*(15.4)
		*Preference*	*2*	*0*	*2(1.6)*
	Nipple shield	Once only	1	1	2
	n = 17	Up to 5 times	4	3	7
		More than 5 times	4	4	8
		*Knowledge*	*32*	*55*	87*(70.7)
		*Preference*	*9*	*5*	*14(11.4)*

*n = 128, remainder did not indicate knowledge of the strategy.

### Knowledge and preference

Knowledge and preference was noted for all respondents. For each second-line strategy women indicated whether or not they previously knew of the strategy, and if not, to indicate if they would prefer to have known. As shown in Table 2, a high proportion of women already knew of bottles with regular and wide teats, and nipple shields. Variants of syringe feeding, cup, and supply line strategies were less well known. With the exception of nipple shields, women indicated very little preference for strategies they did not have previous knowledge of.

### Key challenges

The seven key challenges cited by women for using second-line strategies are shown in Table 3. More than one third reported ‘nipple pain’ (n = 25), ‘baby would not settle’ (n = 25), ‘not enough breast milk or colostrum’ (n = 23), and ‘fatigue’ (n = 23). The category ‘baby could not latch – other reasons’ (n = 23), contained eight challenges to breastfeeding including uncoordinated suck, breast refusal, flat and/or inverted nipple shape, engorgement, and jaundice.

**Table 3:** Key breastfeeding challenges and parity

Key challenges * (%)		**n** = **62 (%)**	**Primipara**	**Multipara 2, 3, 4**
	Nipple pain	25 (40)	14	11
	Baby would not settle	25 (40)	13	12
	Not enough breastmilk or colostrum	23 (37)	16	7
	Fatigue	23 (37)	14	9
	Night nursery	16 (26)	9	7
	Permission			
	Baby lost 10% or more	15 (24)	9	6
	Birth weight			
	Pain associated with	13 (21)	9	4
	Birth			
	Baby could not latch	23 (37)		
	Various reasons**			
**Fatigue** (n = 23) (37)				
	Mild	4 (17)	3	1
	Moderate	6 (26)	2	4
	Strong	7 (30)	5	2
	Extreme	6 (27)	4	2
**Nipple pain** (n = 25) (40)***				
	Mild	1 (5)	1	0
	Moderate	4 (20)	3	1
	Strong	10 (50)	6	4
	Extreme	5 (25)	3	2
**Birth related pain** (n = 13) (21)****				
	Mild	0 (0)	0	0
	Moderate	5 (42)	3	2
	Strong	5 (42)	3	2
	Extreme	2 (17)	1	1

*respondents could choose more than one.

**engorgement, flat or inverted nipples, sleepy (hypoglycaemia, jaundice), disorganized suck.

***missing data = 5.

****missing data = 1.

### BFSE

The overall BSES-SF mean was 51.18 (SD = 12.48). The mean BSES-SF score of women using a second-line strategy was 43.43 (SD = 12.19), and of women not using a second-line strategy it was 58.32 (SD = 7.40). Mean BSES-SF scores, parity, and Kruskal-Wallis analysis of median scores are reported at Table 4. The median BSES-SF score was 53.00. Sixteen women using second-line strategies exceeded the median, and 42 women scored less than the median (p = 0.001). Forty-four women not using second-line strategies scored above the median, and nineteen women scored below it.

**Table 4:** BSES-SF Scores and parity, Kruskal-Wallis

BSES-SF scores* n = **121**		**Whole sample (SD)**	**Did not use any second-line strategies (SD)**	**Used one or more second-line strategies (SD)**
Mean	Whole sample	51.18 (12.48)	58.32 (7.40)	43.43 (12.19)
	Primiparous		55.06 (6.65)	41.00 (12.30)
	Multiparous		59.43 (7.37)	47.13 (11.29)
Median		53.00		
	> median		44	16
	≤ median		19	42
Kruskal-Wallis ANOVA	H statistic	p-value		
	21.569	0.001		

*(possible range 14–70).

BSES-SF: the Breastfeeding Self-Efficacy Scale - Short Form.

SD: standard deviation.

## Discussion

The first important finding in this study is that mean BFSE levels in the group of women not using any second-line strategies was relatively high (58.32) (43% primiparas). Other studies measuring BFSE at 7 days range from 44.7 (48.5% primiparas) [29] to 55.8 (45% primiparas) [20], however use of second-line strategies is not reported in those studies. This finding is promising, given the important role that BFSE is likely to play in longer term breastfeeding duration [12,13,22]. It should be noted, however, that the sample was recruited from one site, and resulted in a relatively homogenous group of respondents whose age, formal education, and combined household income were consistently higher than average for the Australian population [30]. The women’s professional roles and higher educational levels may have influenced their BFSE. Moreover, breastfeeding history is a mode of mastery experience and a prime source of breastfeeding self-efficacy [19]. The multiparous women in this sample reported high rates of breastfeeding for previous children, with many reporting previous breastfeeding for more than 6 months. Studies have reported that breastfeeding history predicts future breastfeeding initiation and duration [3,25,31], and as such is another factor that may contribute to the higher levels of BFSE in this study. However, while the results of this study highlighted BFSE levels could be associated with longer duration of breastfeeding for the sample, there was significant variation in women’s responses. Considering the limitations of sampling and design the results cannot be widely generalized, however, the findings are an important reminder that individualized assessment and intervention is required to support breastfeeding women.

A further important finding in this study is that women had extensive knowledge of second-line strategies, and overwhelmingly had very little preference to use one other than those they did actually use. The exception to this was nipple shields. The preference for nipple shields is consistent with other studies showing that women are satisfied with their use of nipple shields, indeed, found them indispensible in aiding to achieve breastfeeding goals, and would use them again in the future [31-35]. Nipple shields are unique amongst the repertoire of second-line strategies by facilitating almost direct breastfeeding. Such responses suggest women are possibly demonstrating a desire for knowledge and access to nipple shields, as they may enable them to retain a greater degree of control of the breastfeeding experience when challenges have arisen.

### Key challenges

The seven key challenges cited by women using second-line strategies in this study are consistently noted in the literature [2,7,16,17,36-38]. One of the most common challenges was nipple pain, with others being infant behaviour (‘baby would not settle’), perceived insufficient breast milk supply (‘not enough breast milk/colostrum’), and fatigue. The latter three suggest an interplay of challenges to breastfeeding occurring at night. This is consistent with one Canadian study reporting a high risk factor for supplementary feeding to be time of birth (7 pm to 9 am) [36]. In that study nurses reported breastfeeding problems, infant behaviour and maternal fatigue were reasons for supplementation also. Given that frequent feeding at night by the second/third night is considered normal [39], birthing facility practices such as lower level of staff at night may be an important factor that has been overlooked by breastfeeding advocates.

### Implications

The Baby Friendly Health Initiative (BFHI) Ten steps to successful breastfeeding step nine is to “give no artificial teats or pacifiers to breastfeeding infants” [10]. Almost all the women in the sample who began breastfeeding continued to breastfeed at day seven, despite a high proportion of bottle and teat use. Although bottle and teat use, whether containing breast milk or non-breast milk is widely viewed as harmful to breastfeeding success, this has been challenged in the literature [1,39,40]. In this study more than half the second-line strategies used were BFHI preferred (non artificial teat).

Woman centred care revolves around the guiding principles of control, choice, and continuity [41]. In many settings breastfeeding education and guidance is a part of care provided by midwives. This key component of midwifery practice should therefore be based on the principles of woman centred care. That is, control of breastfeeding aspects in the first week postpartum including decision-making if challenges occur. At present in Australia, second-line strategies are often controlled by policies at birthing facilities [42,43] and opportunities for women to engage in informed decision making about such strategies may be limited. There is some discussion in recent literature that control of breastfeeding should be ‘handed back’ to women [41,44,45]. Evidence of the success of this approach has been provided by Wan et al. [46], who found increased satisfaction in woman-centred postpartum care rather than task-centred postpartum care. In one Swedish study, Zwedberg and Naeslund [47] argue that the current midwifery approach was not entirely woman-centred. Instead they note care models can be family centred, infant-centred, and mother/infant dyad centred. They noted that midwives viewed the woman/midwife relationship from both subjective and objective viewpoints, with the objective viewpoint resulting in woman-centred care being impossible. Just as women birth their infants, not the attendants present at the birth, “the individual woman is responsible for the success of breastfeeding” [44]. Choice of second-line strategies by the woman should be based on her own values and beliefs (which form BFSE via vicarious learning and verbal/social persuasion), establishing a continuance of woman-centred care beyond birth. Where women have knowledge deficits, a continuity of care model facilitates education based on midwives prior knowledge of the woman [45].

## Conclusions

The sample in this study described high rates of positive obstetric practice, such as skin-to-skin contact at birth and positive breastfeeding experiences. A notable proportion of women also reported use of second-line strategies. Importantly, the women using second-line strategies had significantly lower BFSE than those who did not use these strategies. The complex matrix of factors contributing to BFSE requires the implementation of woman-centred principles to breastfeeding and postpartum care which include the informed choice and control of second-line strategies, and continuity of care. Further research investigating woman-centred care for breastfeeding women would be valuable.

## Abbreviations

BFSE: Breastfeeding self-efficacy; BFHI: Baby friendly health initiative; BSES: Breastfeeding self-efficacy scale; BSES-SF: Breastfeeding self-efficacy scale – short form; SPSS: Statistical package for social sciences.

## Competing interests

The author declares that she has no competing interests.
